# How useful is pre-referral pediatric spine imaging?

**DOI:** 10.1007/s43390-023-00687-w

**Published:** 2023-04-15

**Authors:** Dorothy J. Kim, Jennifer A. Dermott, Andrew W. Howard, David E. Lebel

**Affiliations:** Hospital for Sick Children, 555 University Avenue, Room S229, Toronto, ON M5G 1X8 Canada

**Keywords:** Idiopathic scoliosis, X-rays, Diagnostic imaging, Cobb angle, Accuracy

## Abstract

**Purpose:**

Primary care physicians rely on radiology reports to confirm a scoliosis diagnosis and inform the need for spine specialist referral. In turn, spine specialists use these reports for triage decisions and planning of care. The objectives were to determine (1) the adequacy of index images to inform treatment decisions at the initial consultation and (2) the utility of index radiology reports for appropriate triage decisions.

**Methods:**

A retrospective chart review was conducted including all idiopathic scoliosis patients seen for initial consultation, aged three to 18 years, between January 1 and April 30, 2021. A score out of five was generated based on the adequacy of index images to provide accurate Cobb angle measurements and determine skeletal maturity. Index images were considered inadequate if repeat imaging was necessary. Index radiology reports, associated imaging, and new imaging, if obtained at the initial consultation, were compared.

**Results:**

Of the 94 patients reviewed, 79% (*n* = 74) required repeat imaging at the initial consultation, of which 74% (*n* = 55) were due to insufficient quality and/or limited field of view. Of index images available for review at the initial consult (*n* = 80), 41.2% scored five out of five, and 32.5% scored two or below. Comparing index radiology reports to initial visit evaluation with ≤ 60 days between imaging (*n* = 49), discrepancies in Cobb angle were found in 24.5% (95% CI 14.6, 38.1) of patients. The Risser stage was reported in 14% of index radiology reports.

**Conclusion:**

Although pre-referral pediatric spine radiographs serve a diagnostic purpose, most are inadequate for comprehensive idiopathic scoliosis evaluation.

**Level of evidence:**

III.

## Introduction

Idiopathic scoliosis (IS) is a structural deformity of the spine characterized by lateral curvature and vertebral rotation, typically affecting 4% of the pediatric population [1]. The severity of the scoliosis is quantified radiographically by the Cobb angle, and in combination with skeletal maturity indicators, guides triage and treatment considerations [2, 3]. The current Scoliosis Research Society (SRS) guidelines for brace treatment are for IS patients with curves between 25 and 40° and with a Risser score of 0–2, indicating meaningful growth remaining. Surgical intervention is reserved for patients with curves larger than 50° and observation is recommended for those with curves less than 25° [4]. Brace treatment is known to minimize the risk of curve progression to the surgical range in skeletally immature youth [5]; therefore, there is an impetus to diagnose this group of patients early to minimize the number of avoidable spine surgeries. In part, this is contingent on the referral from the primary care physician (PCP) and thus the quality of pre-referral spine radiographs and the associated radiology report.

Without nationwide screening programs for scoliosis, PCPs are often the first point of contact for patients and are tasked with ordering imaging when scoliosis is suspected [6, 7]. They rely heavily on radiology reports to confirm a clinical IS diagnosis and inform the need for spine specialist referral. These radiology reports are used by clinic staff to inform triage decisions and to allocate finite clinic resources, ensuring time-sensitive referrals are prioritized, specifically potential brace candidates. To ensure these patients can be properly identified, it is essential that index imaging captures the radiographic features necessary to measure Cobb angle and assess skeletal maturity, and that these results are accurately reported.

The specific objectives of our study were to evaluate (1) the utility of index radiology reports for appropriate triage decisions and (2) the adequacy of index spine images to inform treatment decisions at initial spine specialist consultation.

## Methods

### Study design

We conducted a retrospective chart and radiographic review of all idiopathic scoliosis patients between 3 and 18 years of age seen for an initial consultation, from January 1-April 30, 2021. Patients with non-idiopathic etiologies and those seeking a second opinion were excluded. Formal consent was not required for this study. The proposed project underwent independent review and was approved by our institution’s central Quality Improvement Department (QIP-2021-04-29T09-08-04).

Comparisons were made between the index radiology report, its associated imaging, and new imaging, if obtained at the initial consultation. Measurements performed by a spine specialist were considered the gold standard. Comparisons between the index report and the gold standard were made only when there were less than 60 days between imaging, a duration too short to be expected to capture actual progression [8]. Discrepancies in the measurement of the major curve (largest Cobb angle) between the index radiology report and the gold standard were calculated.

### Outcomes

Baseline data included sex and age at initial presentation, date and location of index imaging (hospital or community-based private clinic), the largest Cobb angle noted in the radiology report or PCP referral, as well as any documentation of skeletal maturity indicators. When index imaging was available, the Cobb angle was remeasured by one of the authors. If imaging was repeated at the initial consultation, the spine specialist’s measurement of the largest Cobb angle and Risser stage was recorded. Treatment decisions and planning were based on SRS guidelines [4].

### Image quality

A quality score out of five was generated based on the adequacy of index images to provide accurate Cobb angle measurements and determine skeletal maturity (Table 1). The coronal view was weighted with more significance given its necessity for IS diagnosis and Cobb angle measurement and assigned 2-points. Digitally stitched and unstitched images were assigned 1- and 0-points, respectively, to capture attributable inaccuracies. Visualization of the pelvis was assigned 1-point for its utility to evaluate skeletal maturity through tri-radiate cartilage and iliac apophysis fusion. The lateral view was assigned 1-point for the ability to evaluate the sagittal profile of the spine.

**Table 1:** Image quality scoring legend

Feature	
Full spine, coronal view	2
Full spine, lateral view	1
Pelvis	1
Ribcage	1
Total score	5

Index images were considered inadequate if repeat imaging was necessary at the initial visit within 4 months of index imaging, sooner than would be recommended [9]. The reason(s) for repeat imaging was recorded as the following: (i) to obtain a lateral view, (ii) to obtain the view of pelvis, (iii) to obtain images necessary for comprehensive evaluation of the thorax (e.g., ribcage visualization), (iv) poor quality (e.g., non-stitched spinal images) and (v) other.

### Report accuracy

Major discrepancies were defined by inter-reader difference ≥ 15° of the same image, discordant Risser staging impacting growth predictions, or inaccuracies that led to inappropriate triage decisions. Knowing that the average curve magnitude at initial presentation is 37° [10], it is reasonable that discrepancies ≥ 15° lead to inappropriate triage as possible surgical candidates may not be identified.

Triage was considered appropriate if the patient’s predicted treatment (e.g., brace candidate) corresponded with their actual treatment plan after the initial consultation. The location of index imaging, hospital versus community-based private clinic, was evaluated as a risk factor for inadequate or discrepant imaging.

### Statistical analyses

Descriptive statistics were used for patients’ demographic profile. Frequencies were calculated for categorical data. Chi-squared tests were performed on categorical data and the odds ratio using the Wald method was calculated [11]. Confidence intervals of proportions were calculated using the Wilson method [12].

Statistical significance was accepted at a *p*-value less than 0.05. Data were analysed using the web-based stats program R (http://www.R-project.org).

## Results

**Fig. 1:**
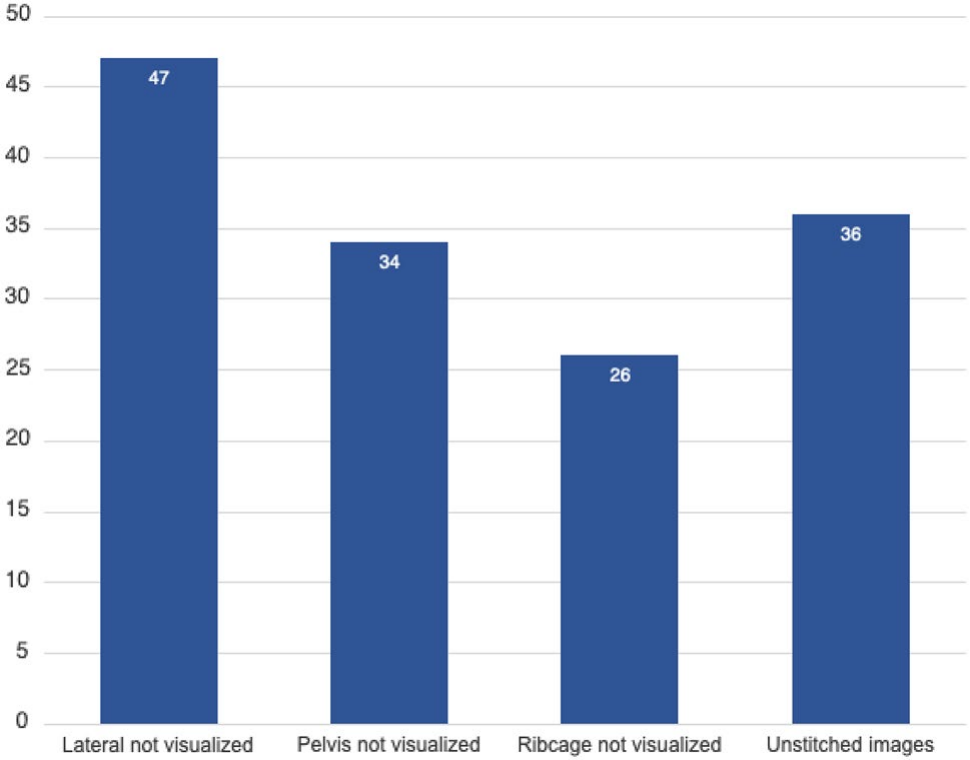
The qualities of pre-referral imaging necessitating re-imaging at initial consult for 74% of patients

There were 94 consecutive patients who met the inclusion criteria over the 4-month period. The patient cohort was typical of an IS population: mostly female (*n* = 73) with an average age of 14.1 years. All patients had an index radiology report, and 85% (*n* = 80) had the associated index image available for specialist review at the time of the initial consult. Seventy-nine percent (*n* = 74) of patients required repeat imaging at the initial consultation, of which 74% (*n* = 55) were due to insufficient quality and/or visualization of the sagittal profile, pelvis, or ribcage (Fig. 1).

Quality scores showed 41.2% (*n* = 33) scored five out of five and 32.5% (*n* = 26) scored two or below. New imaging obtained at the initial consultation showed that 50.0% (*n* = 13) of patients with scores two or below were not triaged appropriately. For example, their initial visit was with a non-surgical health care provider anticipating they were a brace candidate; however, their curve was in a surgical range. Comparatively, 81.2% (*n* = 27) of patients with a full score were triaged correctly.

The Risser stage was reported in 14% (*n* = 13) of index radiology reports, of which 77% (*n* = 10) originated from a hospital radiology department.

Re-evaluation of index imaging (*n* = 80) by a spine specialist demonstrated that 24.6% (95% CI 15.2, 37.1) of Cobb angles were misreported by more than 5° (range 6–21°). Comparing index radiology reports to initial visit evaluation with ≤ 60 days between imaging (*n* = 49), discrepancies in Cobb angle were found in 24.5% (95% CI 14.6, 38.1) of patients and 18.4% (95% CI 10.0, 31.4) were categorized as major discrepancies (Table 2). In 13.8% (*n* = 13) of the total cohort, surgical or brace treatment was recommended at initial consultation when not predicted based on the index radiology report.

**Table 2:** Types of discrepancies, pre-referral imaging versus initial consult imaging

Type of discrepancy	Number of patients (*n* = 49)
Cobb angle discrepancy 6–10°	8
Cobb angle discrepancy 11–15	0
Cobb angle discrepancy > 15^a^	3
Risser staging reported 5, actual 0^a^	1
Treatment change: predicted brace, actual surgery^a^	3
Treatment change: predicted observe; actual brace^a^	2
Treatment change: predicted brace; actual observe	4

^a^Denotes major discrepancy

**Fig. 2:**
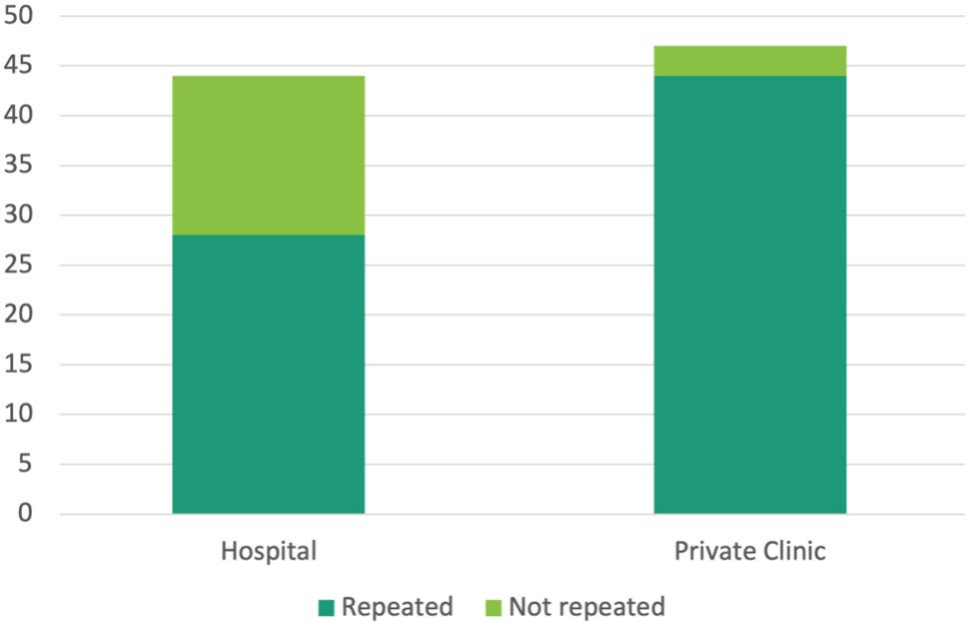
Frequency of repeat imaging at initial consult was dependent on location of pre-referral imaging. Repeat radiograph was more likely (OR 8.38, *p* = 0.001) when index imaging was obtained at community-based private clinic

**Fig. 3:**
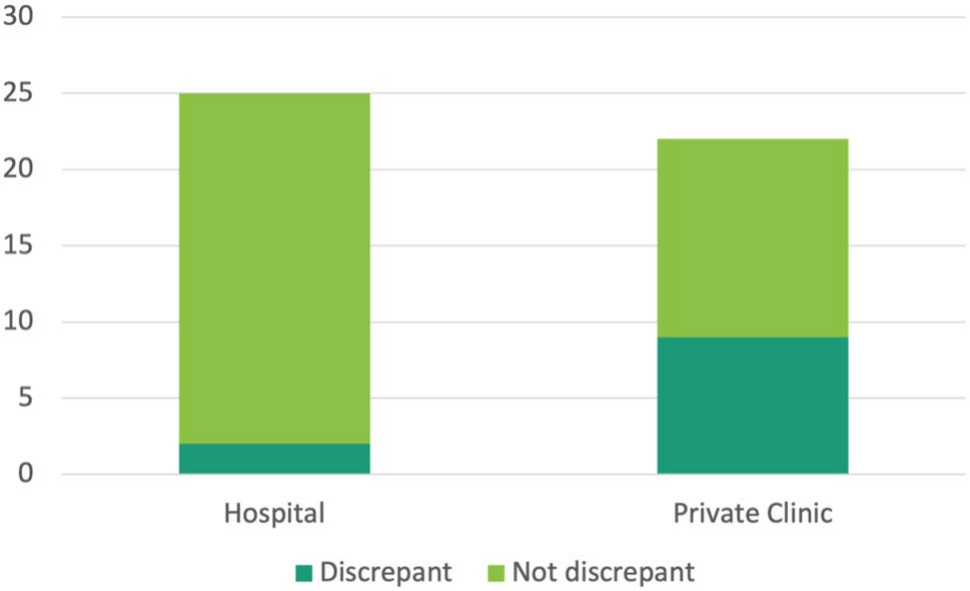
Frequency of Cobb angle discrepancies was dependent on location of pre-referral imaging. Cobb angle reported on index imaging was found to be discrepant more often (OR 7.96, *p* = 0.02) when index imaging was obtained at a community-based private clinic

Images were obtained in community-based private clinics (*n* = 47), at a hospital (*n* = 44) and in undisclosed locations (*n* = 3). Repeat radiographs (OR 8.38, *p* = 0.001) and discrepancies (OR 7.96, *p* = 0.02) were increased when index imaging was obtained at a community-based private clinic compared to at a hospital (Figs. 2 and 3).

## Discussion

The quality of index spine radiographs as well as the accuracy and detail of the associated radiology reports are key components in the IS referral process from primary care to specialized spine clinics. Appropriate triage is jeopardized when index imaging is inadequate, or reporting is inaccurate. The 5-point scoring system which we used for index images is based on the 3-foot standing spine PA and lateral X-ray—the gold standard for comprehensive scoliosis evaluation [3]. In our study, half of the patients with index imaging scoring 2 or less, were triaged inappropriately leading to inefficiencies in our effort to optimize access to treatment. Furthermore, discrepancies were increased when index imaging was obtained at a community-based private clinic compared to at a hospital. To best ensure its value, and minimize unnecessary repeat exposure to radiation, index imaging should be adequate for triage and clinical decision-making. As such, we recommend that index spine imaging visualizes the entirety of the trunk and pelvic skeletal system.

### Components of comprehensive spine X-ray

#### Full spine visualization

A field of view that includes the entire spine in a single image facilitates accurate measurement. Many facilities digitally stitch images together to render a “full” visualization; however, digital stitching errors are reported with 16% of errors that could result in false diagnoses [13]. Accuracy is further hampered when images remain unstitched, particularly if the curve is not contained in a single image. In our study, when compared to a spine specialist’s measurements, nearly one-quarter of index radiology reports misrepresent the Cobb angle by more than 5°. While small differences in measurements may be clinically insignificant, inaccurate results are meaningful when they affect triage and timely access to treatment. Report inaccuracies may result in mild curves being accepted and unnecessarily occupying clinical spots, or more importantly, if under-estimated, moderate curves may be rejected and the opportunity for conservative treatment may be lost. During the study period, 14% of patients (*n* = 13) had alternate treatment than what was anticipated. Radiology reports were inaccurate to the extent that brace treatment and surgical intervention could not be reliably predicted. Inaccuracies not only contribute to suboptimal triage decisions, but undue stress and sometimes a sense of distrust is experienced by patients and their families when the information provided by their PCP, informed by the index radiology report, does not resemble what they are messaged at their initial clinic consult.

### Pelvis visualization

The pelvis must be fully visualized to determine if the triradiate cartilage is fused or not, and to assess Risser staging as these measures are instrumental in determining skeletal maturity. The Risser staging system is the most frequently used method of evaluating skeletal maturity and is a vital part of scoliosis evaluation [14]. However, our study shows that only 14% of radiology reports include Risser signs or mention ossification of the iliac apophysis. The status of the triradiate cartilage, fused or unfused, was not mentioned in any report. While shielding is one reason that the pelvis may be obscured in spine images, there is growing scientific evidence that shielding provides negligible or no benefit and carries a substantial risk of increasing the patient’s radiation dose and compromising the diagnostic efficacy of an image [15, 16]. In the absence of a documented Risser sign, triage decisions related to growth potential are then informed by chronological age and menarchal status, if applicable and accurately provided by the referring physician. However, with the considerable variability in peak height velocity as it relates to both age and menarchal status, inaccurate assumptions may be made about skeletal maturity from referral intake data alone [17, 18]. A Cobb angle magnitude without the context of skeletal maturation offers little insight into the prognosis of spine deformity. Identifying patients between Risser stages 0 to 2 helps to prioritize those at the highest risk of curve progression, who need to be seen as soon as possible.

### Lateral view

A lateral view of the spine was the most common aspect missing from index imaging necessitating repeat imaging within a short time interval. Nearly, three quarters of patients required re-imaging prior to the recommended 6-month interval as their index imaging was inadequate for comprehensive evaluation [9]. It is our opinion that the sagittal profile of the spine must be assessed as abnormalities in this plane can be indicative of non-idiopathic etiologies, or reveal a spondylolysis or -listhesis, or end-plate irregularities [19, 20]. Moreover, the two most common reasons that PCPs order spine X-rays are (1) a positive clinical screen and/or (2) reported back pain. Performing only a coronal scan insinuates that the scoliosis is explanatory for back pain when, in actuality, spine pathology that would result in back pain is more likely to be found in the sagittal plane.

Rampersaud analyzed health care costs and utilization for adult spinal conditions in Ontario (Canada) and determined that spinal imaging costs were $66.5 million per year with 35.6% attributed to spine X-rays [21]. By repeating radiographs so frequently, not only is there a burden of cost to the overall health care system, but there is also a significant risk of over-radiation to the individual patient. A recent systematic review and meta-analysis of over 35, 000 patients demonstrated that patients with adolescent IS are at increased risk of cancer and mortality related to cancer due to repeated radiographs [22].

### Limitations

The limitations of this study include the methodological shortcomings of a retrospective review. Not all index imaging was available for review in our Picture Archiving and Communication System (PACS) if obtained at a private community clinic that does not employ Secure File Transfer Protocol (SFTP). Although the short study period may represent a snapshot in time, the results are consistent with our clinical experience. We found that imaging obtained at a hospital was significantly more accurate and adequate than that obtained at a private community clinic. The results, however, may have been skewed by including images obtained at our institution, the only facility within this study having a pediatric spine specialty clinic. Nevertheless, this subset of hospital index images is a small number, and the results would still have been significant. Accuracy of Risser, when included in a radiology report, was not assessed in this study. Only 13 reports mentioned Risser staging but if there is a mandate to include this measure in standardized reporting, the accuracy of what is being reported must be examined in the future.

The results of this study precipitate further study into ways to improve the accuracy of Cobb angle measurements, such as the use of artificial intelligence and the application of machine learning to interpret spine X-rays, and the need for standardization of spine imaging and reporting.

## Conclusion

Other than providing basic diagnostic confirmation of scoliosis, the majority of index spine X-rays are inadequate to provide a comprehensive evaluation of idiopathic scoliosis, particularly if obtained at a community-based private clinic. Improving the quality and accuracy of spine imaging and reporting is critical to facilitate appropriate triage and timely treatment as well as to decrease unnecessary radiation exposure and public healthcare costs. A study to further examine the diagnostic validity of the community spine radiograph for AIS and its potential impact on early referral and management will be important.

## Data Availability

The datasets generated and analyzed for the current study are available from the corresponding author upon reasonable request.
